# Secretory Products of *Trichinella spiralis* Muscle Larvae and Immunomodulation: Implication for Autoimmune Diseases, Allergies, and Malignancies

**DOI:** 10.1155/2015/523875

**Published:** 2015-05-31

**Authors:** Ljiljana Sofronic-Milosavljevic, Natasa Ilic, Elena Pinelli, Alisa Gruden-Movsesijan

**Affiliations:** ^1^Institute for the Application of Nuclear Energy (INEP), University of Belgrade, Banatska 31b, 11080 Belgrade, Serbia; ^2^Centre for Infectious Disease Control Netherlands, National Institute for Public Health and the Environment (RIVM), P.O. Box 1, 3720 BA Bilthoven, Netherlands

## Abstract

*Trichinella spiralis* has the unique ability to make itself “at home” by creating and hiding in a new type of cell in the host body that is the nurse cell. From this immunologically privileged place, the parasite orchestrates a long-lasting molecular cross talk with the host through muscle larvae excretory-secretory products (ES L1). Those products can successfully modulate parasite-specific immune responses as well as responses to unrelated antigens (either self or nonself in origin), providing an anti-inflammatory milieu and maintaining homeostasis. It is clear, based on the findings from animal model studies, that *T. spiralis* and its products induce an immunomodulatory network (which encompasses Th2- and Treg-type responses) that may allow the host to deal with various hyperimmune-associated disorders as well as tumor growth, although the latter still remains unclear. This review focuses on studies of the molecules released by *T. spiralis*, their interaction with pattern recognition receptors on antigen presenting cells, and subsequently provoked responses. This paper also addresses the immunomodulatory properties of ES L1 molecules and how the induced immunomodulation influences the course of different experimental inflammatory and malignant diseases.

## 1. Introduction

*Trichinella spiralis* (*T. spiralis*) is the first identified and best characterized member of* Trichinella* genus. This happened for the following reasons. First, this worm is of an importance as a cause of the human disease, trichinellosis. Second, infection with this helminth has high prevalence in many different sylvatic and domestic animals. And last but not least, its high infectivity for laboratory animals provides valuable* in vivo* models for basic biological, pathological, and immunological studies [1].

Among the different helminths,* Trichinella spp.* are unique because all three life cycle stages of the parasite, infective muscle larvae, adult, and new born larvae, develop in one host. Infection is acquired by consumption of infected raw or undercooked meat. Under the influence of gastric juice, larvae are released in the stomach, molt, and develop into the adult stage inside the enterocytes of small intestine. After mating, new born larvae are released into circulation and spread throughout the tissues and organs and only those that enter striated muscles mature into muscle larvae. Intracellular localization of* Trichinella spp.* takes place at two different tissue sites, namely, in enterocytes and skeletal muscle cells which represent the habitat for this parasite [2].* T. spiralis* has a unique ability to make itself “at home” by transforming the infected muscle cell and creating a new type of cell in the host body, the so-called nurse cell [3, 4]. From that immunologically privileged place, parasite achieves long-lasting communication with the host through muscle larvae excretory-secretory products (ES L1).

In humans, infection could remain asymptomatic if it involves a low number of larvae, but in case of ingestion of few hundred larvae, gastrointestinal symptoms appear as soon as 2 days p.i. followed by development of serious, rarely fatal, disease [5]. Clinical signs of the disease usually last 4–6 months, rarely longer (up to 2 years). It is still debatable whether a chronic form of trichinellosis exists [6] and whether infective larvae remain in striated muscles for years, although specific antibody responses could be detected even 30 years after infection [7]. Host invasion by* Trichinella* larvae induces a complex immune response, which in human is better characterized by humoral rather than cellular responses (due to the importance of humoral response for diagnostic purposes) [2, 8]. Unlike the case in humans,* T. spiralis* can reach in animals a high worm burden without causing clinical symptoms [1].

During the intestinal phase of infection, the immune response involves both Th1 and Th2 responses. Initially Th1 responses are induced followed by a dominant Th2 type of response, characterized by the production of high levels of cytokines IL-4, IL-5, IL-9, IL-10, and IL-13, as well as immunoglobulin E (IgE) and the mobilization of eosinophils, basophils, and mast cells [2]. The muscle phase of the infection is further characterized by the existence of Treg cells. It is likely that chronic stimulation through ES L1 released into the circulation during the muscle phase of* T. spiralis* infection can activate regulatory network elements as guardians of homeostasis [9]. Immune events orchestrated by Th2 and Treg cell types can successfully modulate the immune response of the host [10]. This parasite has evolved to suppress the host immune response against itself in order to survive [2], but it also suppresses immune responses to autoantigens and allergens [11, 12] and prevents or attenuates malignant cell development and expansion [13]. This review summarizes the current knowledge on the mechanisms of immunomodulation engaged by this nematode through the ES L1.

## 2. ES L1 Antigens of* T. spiralis* Muscle Larvae

*T. spiralis* completes its whole life cycle in one host, influencing the host organism with components from each life cycle stage. However, since the establishment of the infection depends on the invasion of intestinal epithelium by infective muscle larvae (ML) and the maintenance of parasitism of the ML in muscle cells, it is clear why the majority of studies focus on the proteins derived from the ML. ES L1 originate from stichocyte granules in the stichosome, secretory organelle of the* T. spiralis* ML [14]. These products participate in the interaction with various host cells such as enterocytes, muscle cells, and immune cells, thus achieving their role in parasitism and immune response induction and modulation [10, 15]. Through ES L1 the parasite creates an environment suitable for its own survival, by either modulation of host immune response or influencing host cell gene expression. The study of these molecules is central for understanding the mechanisms of successful parasitism and also for the development of novel therapies and preventive treatments for inflammatory diseases.

Studies on ES L1 have shown that these are composed of 13 different proteins, although the number of peptide spots, identified with 2-DE and proteomic analysis, was 43 [16]. Obtained data suggest existence of several isoforms of the proteins, which could be the consequence of posttranslational modification (e.g., glycosylation), splice variants, and protein processing. The fact that ES L1 proteins have been shown to have N-terminal signal peptide confirms that they are secretory proteins. Numerous ES L1 proteins are highly glycosylated. Detailed analyses have revealed that these glycoproteins bear multiantennary* N*-glycans composed of GalNAc*β*1-4GlcNAc, capped with tyvelose (3,6-dideoxy-D-arabinohexose) (tyv). The majority of antennae are also fucosylated on the GlcNAc residues. GlcNAc is highly represented in* T. spiralis* N-glycans, where it is either in terminal position or it is implicated in the so-called “lacdiNAC” (GalNAc-*β*1,4-GlcNAc) antennae [17, 18]. Milcheva [18] and the coworkers discovered for the first time O-linked glycans on the surface of* T. spiralis* intestinal and muscle larvae. These structures were analogous to Tn-antigen (GalNAc-*α*-Ser/Thr) and T-antigen (Gal-*β*1,3-GalNAc-*α*-Ser/Thr) and also similar to A-blood group antigens (GalNAc-*α*1,3-Gal-*β*1,3(4)-(Fuc-*α*1,2-)-R). Our results of lectin-blot analyses [19] were in line with previous findings that N-linked glycans of* T. spiralis *muscle larvae contain high mannose-type structures and those with a trimanosyl core, with or without core fucosylation.

The scarce research on the role of individual glycoprotein components of* T. spiralis* ES L1 indicates that the 45 kDa glycoprotein has inhibitory effects on neutrophil function* in vitro* suggesting its possible involvement in the decrease of inflammatory cells around the encysted parasite during the acute phase of infection [20]. However, the exact function of most of the muscle larva ES proteins still remains to be elucidated. ES L1 of* Trichinella spp.* contain some functional proteins such as proteinases, proteinase inhibitors, heat shock proteins, glycosidases, kinases, phosphatases, endonucleases, MIFs, enolases, and DNA-binding proteins [21], determined mostly according to their structural and sequence similarity to proteins from another species with known function. Since* T. spiralis* ES L1 antigens are localized on the surface of the parasite [22] and in the nuclei of infected host cells [23], a possible role of these molecules may be in invasion of the enterocytes, establishment or maintenance of the nurse cell, and induction of immune response in host organism [24].

### 2.1. The Role of ES L1 in Enterocyte Invasion

*T. spiralis* larvae do not possess specialized organ for mechanical penetration [25, 26] and for that reason it was assumed that the molecules on the surface of the parasite, or excreted-secreted, might be responsible for the initial contact with enterocytes and the penetration into the cell [27]. For some time, a significant role for tyvelose-bearing ES L1 glycoproteins in the intestinal phase of the infection has been acknowledged. These glycoproteins are deposited in the intestinal epithelium during the establishment of a new infection [27]. Tyvelose is an immunodominant epitope, which elicits strong antibody response that protects host organism from reinfection. Anti-tyv antibodies promote rapid expulsion of parasites from the intestine by preventing interaction between parasite and host enterocytes, thus interfering with intestinal niche formation [28]. These findings provided a proof that invasion requires direct contact of the larvae and the enterocytes [29], but the molecules involved were not yet identified. Recently it was discovered that infective larvae, cocultured with intestinal epithelial cells, produce novel proteins and that some of these proteins could be found in these cells [30, 31]. It was not, however, possible before the construction of* T. spiralis* phage display library to screen for larval proteins that could be involved in the interaction with enterocytes* in vitro* [32]. For six* T. spiralis* proteins (Tsp1, calcium-transporting ATPase 2 protein; Tsp4, ovochymase-1; Tsp6, T-complex protein 1 subunit eta; Tsp7, glycosyl hydrolase family 47; Tsp8, DNA replication licensing factor MCM3; Tsp10, nudix hydrolase) the molecular functions were assumed. Out of these six proteins, five have catalytic activity, four have binding activity, and one has transporter activity [32].

ES L1 of* T. spiralis* contain several proteases, such as serine, cysteine, and metalloproteases [33, 34]. Serine proteases found in parasites participate in host tissue and cell invasion and likely play a role in molting of nematodes [35]. Serine proteases were found in* T. spiralis* ES L1 [16, 31, 36, 37] and for some of them collagenolytic and elastolytic activities were detected [38], which suggested their possible involvement in the invasion of intestinal epithelial cells. Gene expression analysis of* T. spiralis* revealed existence of zinc-dependent metalloprotease [39], which might be involved in the process of tissue invasion, by degradation of fibrinogen and plasminogen. Namely, it was found that this zinc-dependent metalloprotease possesses significant homology to the astacin metalloprotease family of* Caenorhabditis elegans*, participating in hydrolysis of type I collagen [40].

### 2.2. The Role of ES L1 in Muscle Cell Transformation

Muscle phase of* T. spiralis* life cycle involves the transformation of the host muscle cell into completely new entity, the nurse cell. Changes in infected muscle cell are dictated by the invading parasite through secreted proteins released into the matrix of the cell; however, the identity of these molecules still awaits being discovered. The majority of data concerning muscle stage of the infection refer to transcriptome analysis of infected muscle cell, that is, the level of expression of genes involved in muscle cell differentiation and reparation. Although it has been assumed that* T. spiralis* ES L1 actively participate in nurse cell formation, the real evidence emerged from* in vitro* studies using C2C12 myoblast cell line, incubated with these products. It was shown that ES L1 promote myoblasts proliferation and at the same time inhibited their differentiation [15]. However, little is known about molecular mechanisms underlying dedifferentiation of the damaged muscle cells and misdifferentiation of the satellite cells after larvae invasion. Gene expression analysis again revealed that myogenic regulatory factors, MyoD and myogenin (important for the myogenesis and regeneration of muscles), are overexpressed in infected muscle tissues during* T. spiralis* infections, and the MyoD factor is highly expressed in the satellite cells of infected muscles [41]. Further studies added more differentiation-associated genes to that list, which were upregulated during nurse cell formation [42]. Some of the homologs of these proteins have been found in* T. spiralis* ES L1, like MyoD-like, helix-loop-helix-like, DNA-binding FYVE finger domain, and Rcd1-like proteins, which suggest their implication in the above-mentioned processes. Newly identified proteins produced by early muscle larvae are speculated to play a role in nurse cell formation [43, 44]. In addition, fusion family proteins produced by* T. spiralis* have been identified to promote cell-to-cell fusion [45]. Nurse cell formation is accompanied with the formation of the collagen capsule [3] and* de novo* angiogenesis mediated by vascular endothelial growth factor (VEGF) [46], but the molecules originating from muscle larvae responsible for these events have not yet been discovered.

### 2.3. The Role of ES L1 in the Immune Response

*T. spiralis* infection elicits strong immune response in host organism through a number of molecules expressed on the surface or excreted/secreted. The particular group of muscle larvae glycoproteins, named* T. spiralis* larvae group 1 (TSL-1), interact with host immune cells and induce immune response that protects host from reinfection and also enables the survival of the parasite in the host organism. TSL-1 antigens are highly glycosylated and characterized by the presence of carbohydrate epitope, tyvelose. This group was identified based on the recognition of tyvelose residues by monoclonal antibodies specific to muscle larvae [47]. TSL-1 antigens are released from the stichosome of the muscle larvae, both in the intestinal epithelial cells and in muscle cells. They consist of at least six glycoproteins bearing tri- and tetra-antennary* N*-linked structures with subterminal* N*-acetylgalactosamine (GalNAc) residues that are *β*-linked to* N*-acetylglucosamine (GlcNAc) [17]. The antennae glycans are fucosylated in their GlcNAc residues and capped with immunodominant epitope 3,6-dideoxy-D-arabinohexose tyvelose or D-tyvelose [48, 49]. These glycoproteins with Mw 40–70 kDa induce the production of specific antibodies that are often used for diagnostic purposes [8, 47]. Results obtained by analyses of anti-TSL-1 antibody presence in sera of infected animals revealed that they specifically recognize antigens of 40–70 kDa from ML homogenates and antigens of Mw 45–55 kDa in ES under reducing conditions [50–52]. Our results showed that, among TSL-1 antigens, 45, 49, and 53 kDa glycoproteins are recognized in Western blot by sera obtained from* T. spiralis*-infected humans, pigs, dogs, horses, and rats [53].

The 43 kDa glycoprotein of* T. spiralis* muscle larvae was first isolated by Gold et al. [54] and further characterized by Su et al. [55] (referred to as the 49 kDa antigen). Genes encoding 43 kDa glycoprotein were found to be expressed by precapsule and postcapsule muscle larvae, which suggest the importance of this protein for capsule formation [56]. Data concerning presence of 43 kDa glycoprotein in the host muscle cells are conflicting. While Vassilatis et al. [57] identified this glycoprotein in nurse cell nuclei, Jasmer et al. [58] failed to do so with antibodies specific for 43 kDa glycoprotein. It is very likely that this glycoprotein is released in the host muscle cell, presumably with a role to transform this cell into the nurse cell [21]. Recently, another role has been assigned for 43 kDa glycoprotein. MacLea et al. [59] proposed deoxyribonuclease II*α* (DNase II*α*) activity on the basis of sequence homology with* C. elegans* DNase II*α*. Similarity of 43 kDa glycoprotein with DNase II*α* was confirmed by a work of Jasmer and Kwak [60] who used myoblasts transfected with plasmids containing gene for 43 kDa glycoprotein.

The gene encoding 45 kDa glycoprotein belongs to a multicopy gene family on DNA and encodes several larval proteins ranging from 40 to 50 kDa. This glycoprotein originates from *β* and *γ* stichocytes and exists in two forms tyvelosylated and non-tyvelosilated. This protein belongs to a family of trypsin-like serine proteases [34].

The gene for the 53 kDa glycoprotein and its complete sequence were reported by Zarlenga and Gamble [61]. The gene encoding the 53 kDa glycoprotein is expressed in postcyst larvae and adult worms [56]. The protein is present only in *β* stichocyte granules, shows heterogeneity in glycosylation, and, like other TSL-1 antigens, bears tyvelose epitope [36]. The exact function of this protein remains elusive, but it is assumed that its role is connected with maintaining parasitism and modulation of immune response [21]. Antibodies present in the sera of* T. spiralis*-infected human and animals always recognize this glycoprotein, pointing to a role in the induction of the immune response [52, 53]. The fact that 53 kDa glycoprotein could be frequently recognized by antibodies in the sera from patients with autoimmune diseases [62] adds to its role in immunomodulation. Data obtained by testing immunogenicity of 53 kDa glycoprotein using monoclonal antibodies indicate that this protein bears epitopes that are specific for* T. spiralis* as well as an epitope shared by all encapsulated* Trichinella* [36, 63]. The antigenicity of the 53 kDa glycoprotein is mainly due to its protein epitopes, unlike antibodies to other TSL-1 antigens that are mainly directed to sugar moieties [63].

## 3. Antigen Presenting Cells and* T. spiralis* ES L1 (Receptors, Signaling, Phenotype, and Function)

Key-players in initiation and polarization of adaptive immune response triggered by* T. spiralis* antigens are antigen presenting cells (APCs), namely, dendritic cells (DCs) and macrophages. The essential role for innate immune cells in encountering the parasite products, that is, ES L1 antigens, has been ascribed to DCs, which recognize pathogen-associated molecular patterns (PAMPs) through different pattern recognition receptors (PRRs) which include the toll-like receptors (TLRs), C-type lectin receptors (CLRs), Nod-like receptors, and RIG-I like receptors [64, 65]. There are only few reports on the effect of* T. spiralis* on TLRs and related signaling pathways. The only available published results on* in vivo* infection [66] show that each* T. spiralis* life cycle stage differently influences TLRs expression on splenic cells and consequently has different impact on host immune regulation. Using* T. spiralis* muscle stage antigens, it was observed that these antigens may modulate the immune response primarily through TLR4 dependent pathway by decreasing the expression of this TLR. It has already been demonstrated that most bacterial antigens increase the expression of various TLRs [67] while the helminth antigens downregulate TLR expression [68, 69].* In vitro* studies with HEK cells transfected with mouse TLR4 (TLR4/MD2-CD14) revealed that ES L1 antigens suppress TLR4-mediated activation when using* E. coli* LPS as the TLR4 agonist [70]. ES L1 antigens also suppressed the expression of maturation markers on DC and their cytokine production induced by LPS. Using other TLR agonists (for TLR1/2, TLR2/6, TLR3, TLR5, TLR7, and TLR9) in the presence or absence of* T. spiralis* ES L1, the same group of authors showed that the expression of surface markers MHC II, CD80, CD86, and CD40, as well as the production of cytokines (IL-1*α*, IL-6, IL-10, IL-p70, and TNF-*α*) by treated DCs, was not altered by ES L1, indicating that the effect of* T. spiralis* muscle larvae metabolic product is restricted to TLR4. We have shown that both TLR2 and TLR4 recognize and bind ES L1 antigens, which lead to the activation of TLR2 and TLR4 on HEK cells (unpublished results). It is not yet clear whether binding of different helminth antigens, including* T. spiralis* ES L1, to TLRs directly activates APC or the cross-linking of antigens with other PRRs on APC is needed. The important role in the induction and fine tuning of the immune response is ascribed to carbohydrate entities on helminth antigens, as well as to the lectin-like receptors that bind them [71]. Among CLRs, DC-specific ICAM-3 grabbing nonintegrin (DC-SIGN) recognizes and binds to fucose or mannose residues and high mannose-type N-glycans [72], macrophage galactose binding lectin receptor (MGL) recognizes terminal *α*- and *β*-linked GALNac residues [73], and mannose receptor (MR) binds mannose containing glycans [64]. Each of them can be involved in binding helminth glycans. As many other helminth antigens,* T. spiralis* antigens are rich in carbohydrate residues. Some of these structures are recognized by MR on macrophages and it was shown that activation of macrophages with ES L1 is partly mediated through this receptor [74]. Among the constituents of* T. spirali*s ES L1 glycans, there are tri- and tetra-antennary structures terminated with Lewis^x^-like trisaccharide that are also capped with the genus specific carbohydrate tyvelose (3,6-dideoxy-D-arabinohexose) [17, 75, 76] to create a tetrasaccharide structure [77]. It is still not known which receptor participates in binding tyvelose, but according to findings from other helminths [78, 79] it can be assumed that ES L1 Lewis^x^-like glycan may be recognized by DC-SIGN and MR. Future investigations will give better insight into the different PRRs involved in binding* T. spiralis* ES L1 and their contribution in the internalization of antigens and consequent signaling process.

After stimulation, DCs undergo a maturation process, that is, phenotypic and functional changes, which lead to their migration to the lymph nodes and to priming T cell response [80, 81]. Upon binding of different helminth molecules to PRRs on APC signaling events are triggered and they include the activation of three major mitogen-activated protein kinases (MAPK): the extracellular signal-regulated kinase (ERK), p38, and Jun N-terminal kinase (JNK). Only a few studies have dealt with the activation of signaling pathways that follows DCs stimulation with* T. spiralis* ES L1. Considering the relevance of ERK and p38 signaling in regulation of immune response [82], one of the studies on phosphorylation status of these pathways upon the stimulation of DCs with ES L1 was performed by Cvetkovic et al. [83], including the evaluation of the role of ES L1 glycans on signaling process. This study has shown that native ES L1 induced intense phosphorylation of ERK1/2 and weak phosphorylation of p38, while ES L1 with changed glycan structure after the periodate treatment attenuated the phosphorylation of both MAPK. The intensity of ES L1-induced phosphorylation of ERK1/2 was similar to the one obtained with LPS, a potent stimulator of DCs maturation that strongly enhanced the phosphorylation of both ERK1/2 and p38. Bai et al. [84] in differently designed experiment have shown that* T. spiralis* ES L1 reduced the phosphorylation of ERK1/2 and p38 induced by LPS, which indicated that ES L1 possess the capacity to interfere with the phosphorylation of signaling pathways provoked by other stimuli and thus change the direction of DC maturation upon activation.

Maturation of APC can be evaluated according to the expression of MHC II and different costimulatory molecules on APC surface. Depending on the acquired maturation status, APCs produce different cytokines and chemokines, which influence the adaptive immune response. The key events, enabling DCs to deliver signals to other cell types and to influence the course of immune response, are the upregulation of MHC II and costimulatory molecules CD80, CD86, and CD40 on the cell surface. Full maturation of DCs, reflected in elevated expression of all above-mentioned surface markers and the production of proinflammatory cytokines, IL-12 in particular, is characteristic for DCs exposed to Th1-induced stimuli, such as LPS [81]. Conversely, DCs exposed to helminth antigens acquire semimature phenotype, characterized by limited or no expression of MHC II and costimulatory molecules, and reduced or suppressed release of proinflammatory cytokines and chemokines that result in polarization of immune response towards Th2 and regulatory type [85]. Several studies have demonstrated that* T. spiralis* ES L1 antigens induce no upregulation of MHC II, minor upregulation of CD86, and moderately increased expression of ICAM1 compared to nontreated cells, which is considered as semimature DCs phenotype. DCs stimulated with ES L1 produce significantly increased amounts of immunoregulatory cytokines IL-10 and TGF-*β* and decreased amounts of proinflammatory mediator IL-12p70, compared to nontreated and LPS-stimulated cells, features that suggest their tolerogenic status [83, 86–88]. Tolerogenic DCs are characterized by the ability to limit the inflammation and regulate the immune system through mechanisms like moderate expression of costimulatory molecules, increased production of immunoregulatory mediators (IL-10 and TGF-*β*), decreased production of proinflammatory mediators (IL-12p70), and increased expansion of regulatory T cells [89]. It was already shown that the production of IL-10 and IL-12 by DCs is reciprocally regulated [90, 91] and that IL-10 has a role in downregulation of IL-12 production and in suppression of DCs surface markers expression.

In a separate study Langelaar et al. [92] reported that* T. spiralis* ES L1 did not affect the expression of MHC II, CD80, CD86, and CD40 on the surface of DCs or the production of cytokines by these cells, which may be the consequence of the low amount of ES L1 used in their study compared to others. However, when using this same ES L1 concentration, these authors observed a decreased expression of LPS-induced surface markers on DCs and reduced production of the proinflammatory cytokines IL-12, IL-1, and TNF-*α*, to background levels. The suppressive effect of ES L1 on DC maturation stimulated by LPS was reported to be due to interference of the LPS-TLR4 interaction mediated by parasite antigens. Aranzamendi et al. [70] have also shown the impact of ES L1 on DC maturation and T cell polarization. Findings of Radovic et al. [93] also revealed that ES L1 could alter the maturation status of DCs pulsed with myelin oligodendrocyte glycoprotein (MOG) peptide. ES L1 possess the capacity to modulate cytokine production of DCs previously stimulated with MOG, by suppressing IL-12 and enhancing IL-10 release.

Considering the role of glycans in DCs maturation and function, Cvetkovic et al. [83] have shown that* T. spiralis* ES L1 with altered carbohydrate structures did not affect the expression of surface markers on DCs, or TGF-*β* production, but they resulted in decreased production of both pro- and anti-inflammatory cytokines, that is, IL-12p70 and IL-10. IL-10 is implicated in the induction of Th2 and regulatory responses [94] and since ES L1 with altered glycan structure do not have the full capacity compared to the native ES L1 to promote the release of this cytokine, it can be concluded that ES L1 glycans are important for the induction of the immune response characteristic for* T. spiralis.* Several studies have shown that helminth glycans originating from* S. mansoni*,* A. vitae*, and* T. suis* have an important role in defining DCs phenotype and cytokine production [95–97]. The ability of* T. spiralis* ES L1 to affect T cell proliferation and polarization either through incompletely mature DCs or independently of these cells, both* in vitro* and* in vivo*, will be addressed in the following section.

## 4. T Cell Polarization under the Influence of* T. spiralis* ES L1 Antigens

The active status of DCs can be evaluated, not only on the basis of cytokine production, but also through their capacity to present antigens to T cells and induce their polarization. Although under the influence of* T. spiralis* ES L1 antigens DCs acquire semimature status, they still have the capacity to present ES L1 antigens to T cells and direct their polarization.* T. spiralis* sensitized T cells, isolated from infected animals, vigorously proliferated in response to DCs primed with ES L1, indicating that T cells specifically recognize antigens presented on DCs [83, 87]. When cocultivated with naive T cells, ES L1 pulsed DCs induced 2-3-fold increase in proliferation compared to nonstimulated DCs [83, 86]. The alteration of glycans on ES L1 did not abolish the capacity to induce proliferation, but it did change the intensity of proliferation compared to the one observed with native ES L1 [83].

T cell polarization depends on the type of signals delivered by DCs. Classically TLR activated, fully matured DCs that release an increased amount of IL-12, orchestrated by p38 phosphorylation, promote the development of Th1 type of immune response [81]. In the case of helminths, as Th2 polarizing agents, there is a decrease in IL-12 production as a consequence of the phosphorylation of ERK and stabilization of c-fos transcription factor in DCs [98, 99]. The observed transient ERK1/2 phosphorylation and significantly downregulated release of IL-12, induced in DCs pulsed with* T. spiralis* ES L1 antigens, enable T lymphocytes to be driven into Th2 direction. However the situation in live infection is much more complex, characterized by mixed Th1/Th2 immune response [87], with the activation of regulatory mechanisms reflected in the highly increased production of IL-10 and TGF-*β* [100]. Naive T cells, primed* in vitro* by ES L1-stimulated DCs, did not show the same phenotype induced by* T. spiralis* infection. The effector T cells, induced* in vitro*, produced increased amounts of IL-4, IL-10, and TGF-*β*, with no capacity to produce IFN-*γ* [83, 87]. Thus, ES L1,* in vitro*, act as a Th2/anti-inflammatory polarizing agent of naive T cells. It could also be connoted that ES L1 pulsed DCs population consists of both immunogenic and tolerogenic DCs, which influence T cell polarization toward Th2 and regulatory type. The source of significantly enhanced production of IL-10 and TGF-*β* could be regulatory T cells (Tregs). However, no increase in CD4^+^CD25^+^Foxp3^+^ cells proportion was observed in the resulting population of T cells after* in vitro* priming with DCs pulsed with ES L1, suggesting that either cells, which did not express Foxp3, have something to do with the production of IL-10 and TGF-*β* or for the induction of Tregs other molecules and neighboring cells have to be present [87, 88]. This was confirmed by the study of Ilic et al. [88] that demonstrated the inability of DCs primed with ES L1 to induce* de novo* generation of CD4^+^CD25^+^Foxp3^+^ Treg cells. In contrast to these findings Aranzamendi et al. [70] showed that ES L1 can expand Treg cells* in vitro*. The expanded CD4^+^CD25^+^Foxp3^+^ Treg population was shown to have suppressive activity and to produce TGF-*β*. One of the differences with the above-mentioned study was the animal model used. Aranzamendi et al. [70] used splenocytes derived from OVA-TCR transgenic D011.10 mice that were incubated with OVA- and TspES-pulsed DC. Another difference is the concentration of ES L1 used, which was much less than the one used in the other study. These differences could account for the discrepancies between the results from these two studies. The lower doses of antigens can therefore result in the induction of tolerogenic DCs, which in turn can induce Tregs [101].

*In vivo* experiments revealed that adoptive transfer of ES L1-stimulated DCs into naive recipients generated the immune status equivalent to the one observed during the chronic* T. spiralis* infection [87]. The production of Th1 (IFN-*γ*), Th2 (IL-4), and anti-inflammatory and regulatory (IL-10 and TGF-*β*) cytokines was increased compared to animals that received nontreated DCs. Also, the proportion of CD4^+^CD25^+^Foxp3^+^ T cells among splenocyte population was highly elevated in recipients treated with ES L1 primed DCs similarly to the expansion of these cells during the muscle stage of the infection. While ES L1, via stimulated DCs,* in vivo* induced mixed Th1/Th2 response with the predominance of Th2 and the activation of regulatory response, ES L1 itself, by intraperitoneal application, failed to prime the same immune response. Apparently, in these conditions, ES L1 antigens promote Th2 and anti-inflammatory response, manifested by increased release of IL-4 and IL-10 from spleen cells. However, direct application of ES L1 did not affect the production of TGF-*β* and IFN-*γ* [83]. The same study demonstrated the importance of ES L1 glycans for Th2/anti-inflammatory polarization of immune response. ES L1 with altered glycan structure significantly decreased the release of IL-4 and IL-10 compared to the impact of native antigens on cytokine production. This finding emphasized the significance of carbohydrate entities within ES L1 antigens for the establishment of host-parasite relationship characterized by immune tolerance. Although the application of ES L1 did not remarkably affect the production of TGF-*β*, it induced increased proportion of CD4^+^CD25^+^Foxp3^+^ T cells [93], which corresponds to the effect that ES L1 exerted via DCs [87]. The same study showed that ES L1, applied* in vivo*, exhibited quite peculiar feature, which was the induction of CD4^+^Foxp3^+^ T cells that did not express CD25. High proportion of this type of cells has not been found during infection with* T. spiralis* or after applying ES L1-stimulated DCs. Apparently purified antigens do not induce the same immune response as pulsed DC or infection, but the mechanisms underlying this phenomenon need more extensive investigation.

The data here reviewed points out towards the immunomodulatory properties of ES L1. However, further investigation is needed to reveal which components of the* T. spiralis* ES L1 antigens can modulate DCs function and T cell polarization. The ability of ES L1 to induce tolerogenic DCs favoring anti-inflammatory responses may be helpful in coping with diseases that involve Th1/Th17- or Th2-mediated inflammation, such as different autoimmune and allergic disorders.

## 5. *T. spiralis* Immunomodulation of Autoimmunity, Allergy, and Malignancy

The beneficial effects of* T. spiralis* infection on the course of autoimmune, allergic, and malignant diseases could be the result of the combination of parasite-derived products and parasite-induced immune response [102]. Although it should be borne in mind that* T. spiralis* infection could be followed by adverse effects like downregulation of T cell responses to viral infection, causing its exacerbation [103], it is important to emphasize that Th2 type of immune response induced by helminths may also mitigate tissue damage by reducing harmful inflammation and enhancing tissue repair [104]. Understanding the mechanisms and identifying the molecules that govern complex host-parasite relationship could help us use their properties.

### 5.1. Immunomodulation of Autoimmunity

Inflammatory bowel disease (IBD) is a chronic relapsing inflammatory condition of the gastrointestinal tract that manifests as ulcerative colitis or Crohn's disease [105, 106]. It is accompanied with a Th1 response, marked by elevated levels of inflammatory mediators IFN-*γ* and TNF-*α*, and low levels of IL-4 and IL-10. However, several studies have suggested that cytokine profile in ulcerative colitis includes both Th1 and Th2 cytokines [107] and that the Th1/Th2 balance is important for preventing the development of the disease [108], while other authors have pointed out the role of Th17 in eliciting inflammation characteristic for ulcerative colitis [109]. Khan et al. [110] investigated for the first time the impact of* T. spiralis* infection on the development of colitis in mice, induced by dinitrobenzenesulfonic acid (DNBS) 21 days after nematode infection. This nematode successfully reduced the severity of the disease, indicated by the reduction in the mortality rate and colonic damage. Studies with other helminth infections and other models of colitis indicated that the presence of the parasite suppresses the disease development [111–113]. The proposed mechanisms for the beneficial outcome, obtained in* T. spiralis*-infected mice, were downregulation of myeloperoxidase (MPO) activity, IFN-*γ* expression, and upregulation of Th2 cytokines IL-4 and IL-13 throughout the course of the disease. Instead of inducing inflammatory bowel disease (IBD) in mice previously infected with* T. spiralis*, with established Th2 type of immune response, Zhao et al. [114] used different approach, which implied infection of animals after IBD induction by trinitrobenzenesulfonic acid (TNBS). The results were very promising, since they obtained reduction in mucosal damage, MPO activity, and IL-12 and IFN-*γ* production. Although IBD was already induced,* T. spiralis* infection redirected the mucosal immune system response from Th1 toward Th2 type, enabling amelioration of TNBS-induced disease. acetic acid-induced ulcerative colitis is another model of this inflammatory disease that was used for the investigation of the impact of* T. spiralis* infection. Authors have found that* T. spiralis* infection that preceded the induction of colitis succeeded in inducing disease amelioration, judged by decreased inflammation rate, improved histopathological changes, and decreased mortality [115]. Infection that was performed after colitis induction managed to reduce the severity of the disease, but to much lower extent. Proposed mechanisms were the induction of regulatory responses during the chronic phase of* T. spiralis* infection, reflected in observed elevated proportion of CD4^+^CD25^+^Foxp3^+^ T cells that could tame Th1, Th2, and Th17 responses present in ulcerative colitis.

Motomura et al. [116] focused their investigation on the impact of* T. spiralis* antigens delivery, instead of infection, on colitis development, since administration of live worms carries substantial risk for the recipient organism and therefore cannot be the therapy of choice. Instead of using helminth infection for healing, we could learn from parasites the mechanisms they utilize to survive. Inflammatory mediators, greatly elevated in DNBS-induced colitis, such as inducible nitric oxide synthase (iNOS), MPO, and IL-1*β*, were downregulated in mice treated with* T. spiralis* antigens. On the other hand, application of* T. spiralis* antigens enhanced the production of Th2 cytokine IL-13 and regulatory cytokine TGF-*β*, which are responsible for the suppression of Th1-mediated inflammatory response.

The study of Du and coworkers [117] revealed protective effect of recombinant 53 kDa glycoprotein (rTsP53) in treatment of experimental colitis in mice. Investigation performed with recombinant p53 elucidated its immunomodulatory properties. Namely, immunization of mice by subcutaneous administration of recombinant p53 provoked strong Th2 and regulatory responses and suppressed Th1 response, exerting beneficial effect on TNBS-induced colitis. As a strong antigen, which provoked production of IgG1 in immunized mice, p53 could be considered as a Th2-prone immunomodulator [118]. Recombinant p53 caused reduction in IFN-*γ* and TNF-*α* (Th1 cytokines) and increased production of Th2 cytokines IL-4 and IL-13 in sera of treated mice. At the level of mucosa, expression of TNF-*α* and IL-6 was significantly decreased, while IL-10 and TGF-*β* mRNA were upregulated [117]. IL-10 is an important protective factor to colitis [109, 111]. These authors have also found elevated expression of markers of alternatively activated macrophages (AAM) (Arg-1 and FIZZ1) in the colon tissue. The beneficial role of AAM on the colonic inflammation in mice has been confirmed [119].

Saunders et al. [120] have clearly shown that* T. spiralis* infection delayed onset and modulates the progression of type 1 diabetes (T1D). However, the underlying mechanisms were not so clear. Although Th2 cytokine IL-4 was induced in the presence of* T. spiralis* infection, there was no reduction in the production of IFN-*γ* (Th1 cytokine that accompanies development of type 1 diabetes in NOD mice), which indicates that the activation of Th2 response may not be the only reason for observed alleviation of the disease. IL-10 has been associated with the protection against diabetes [121, 122] and therefore investigated by Saunders et al. [120], as a possible cause of Th1 suppression. However, they have found that* T. spiralis* infection did not increase the production of IL-10 and did not cause inhibition of spleen cell proliferation.

The impact of* T. spiralis* infection or its products on the progression of experimental autoimmune encephalomyelitis (EAE) was extensively investigated. EAE is an animal model of multiple sclerosis (MS), chronic inflammatory, demyelinating, and neurodegenerative disorder of CNS. Combined model of* T. spiralis* infection and EAE in Dark Agouti (DA) rats strongly indicated that infection with* T. spiralis* significantly reduced EAE severity in a dose-dependent manner [123]. The final muscle stage of parasite infection appears to be able to affect the outcome of a particular autoimmune disease in a manner beneficial to the host. The infection was accompanied by increased production of IL-4 and IL-10, and the reduction in IFN-*γ* and IL-17, cytokines crucial for the induction and progression of EAE [100]. The enhanced production of IL-4, Th2-type cytokine, may have protected DA rats from severe illness. Th2 type of response can suppress Th1 immune responses to heterologous antigens, thus showing beneficial effect on the outcome of the autoimmune diseases [124]. Helminth-induced Th2 suppression of autoimmune disease was described in case of T1D modulation by* T. spiralis* infection [120]. IL-10 is a key effector cytokine in EAE resolution [125]. Its role in the modulation of autoimmune diseases was confirmed in investigations involving infection with* S. mansoni* [126] and* H. polygyrus* [127]. Besides being a Th2-type cytokine, IL-10 can also be a product of regulatory T cells. These cells participate in the control of Th1- and Th17-mediated inflammation as well as in the Th2-type response [128] and have a role in the beneficial outcome of EAE [129]. Our study showed that transfer of T cell-enriched population of cells, isolated from* T. spiralis*-infected animals, into uninfected ones before EAE induction, provided a protective effect on the recipients [100]. Transferred cells produced high levels of IL-10 and contained an increased proportion of CD4^+^CD25^+^Foxp3^+^ T cells, which could affect the course of the disease.

Recently, the modulation of EAE was achieved by total soluble protein extract of* T. spiralis* muscle larvae [130]. The impact of* T. spiralis* ES L1 antigens on the induction and the progression of EAE was investigated either via their effect on DCs or by application of ES L1 alone. ES L1-stimulated DCs became tolerogenic and triggered immune response similar to that observed during infection with* T. spiralis* [87]. Application of ES L1-stimulated DCs into recipient rats prior to the induction of EAE resulted in the reduction of clinical signs and duration of illness [131]. Investigation of the underlying mechanisms revealed that ES L1-stimulated DCs altered the immune response responsible for the development of EAE via decreased production of IFN-*γ* and IL-17 and increased production of IL-4, IL-10, and TGF-*β*, as well as through activation of regulatory T cells. Prophylactic application of* T. spiralis* ES L1 ameliorates EAE with the same success as infection did [93]. However, a shift to the Th2-type response in the periphery and in the central nervous system, accompanied by activation of regulatory mechanisms, had a striking, new feature of increased proportion of unconventional CD4^+^CD25^−^Foxp3^+^ regulatory cells both in the periphery and in the central nervous system of animals treated with ES L1 before the induction of EAE.

### 5.2. Immunomodulation of Allergy

The inverse association between helminth infections and allergic disease in humans has been shown in several epidemiological studies [132–134], and using animal models several groups show that certain helminths can reduce allergic responses by eliciting a prominent anti-inflammatory networks involving regulatory T (Treg) cells [135–138]. We have recently shown that, in the chronic as well as in the acute phase of* T. spiralis* infection, mice are protected against experimental allergic airway inflammation (EAAI) and that Treg cells may play a role in this process [139]. EAAI is a well-established mouse model for allergic experimental asthma [140] and in this study mice were infected with* T. spiralis* at different time points in the course of OVA-sensitization/challenge, in order to determine the effect of the different phases of* Trichinella* infection on EAAI. Findings show that BALB/c mice in the chronic phase of* T. spiralis* infection were protected against EAAI as indicated by significantly reduced levels of OVA-specific IgE in serum, decreased levels of Th2 cytokines, and low numbers of eosinophils in bronchial alveolar lavage BAL. Histological analysis of the lungs indicates that mice in the chronic phase of infection had significantly lower OVA-induced peribronchiolitis and perivasculitis compared to mice that were OVA-treated only [139]. In addition, an increased number of CD4^+^CD25^+^Foxp3^+^ cells were observed in spleen of mice with EAAI that were in the chronic phase of infection. This splenic Treg cell population was also found to be suppressive, as shown by suppression assays* in vitro*. Furthermore, transfer of CD4^+^ T cells isolated from chronically* T. spiralis*-infected mice containing higher proportions of suppressive CD25^+^Foxp3^+^ cells to OVA-treated mice conferred partial protection against EAAI, indicating a protective role for these cells [139]. Protection of C57Bl/6 mice against EAAI by chronic* T. spiralis* infection has also been reported by Park et al. [11]. These authors found a significant reduction of macrophages and eosinophils in the BAL of the OVA-challenged animals after* T. spiralis* infection compared to uninfected animals. The airway hyperresponsiveness and IL-5 levels in BAL from OVA-challenged mice after* T. spiralis* infection were significantly lower as compared to the OVA-only challenged mice. The recruitment of Treg cells in lung draining lymph nodes after* T. spiralis* infection was also observed. The authors suggest that Tregs recruited after* T. spiralis* infection might ameliorate lung function and reduce allergic airway inflammation. Humans can be sensitized to aeroallergens early in life and before consumption of* Trichinella*-infected meat; therefore, we carried out an experiment in which mice were* Trichinella*-infected after OVA-sensitization and short before OVA-challenge [139]. By the time the OVA-challenge was given, these mice were in the intestinal or acute phase of infection. Here, partial protection against EAAI was also observed which was restricted to a significant decrease in the levels of eosinophils in BAL and partial reduction of pulmonary inflammation. It has been suggested by Furze et al. [141] that because* T. spiralis* larvae are particularly susceptible to immune attacks the adult worm induces immunomodulatory mechanisms to protect the development of the newborn larvae. The surface antigens and ES products from the adult parasite might act in multiple ways, provoking significant changes in the gut microenvironment which can have broad effects on the immune system, both within and beyond the gut [142]. One of these immunomodulatory strategies might be the induction of Treg cells. During the acute phases of the infection, either before or after OVA-sensitization, an increased number of CD4^+^CD25^+^Foxp3^+^ cells with suppressive activity were observed in the spleen of* Trichinella*-infected mice [139]. The proportion of these Treg cells was however lower compared to the chronic phase of infection. Perhaps, this early involvement of Treg cells may reduce Th2-cell responses, which can explain the observed protective effect against EAAI during the acute phase of* Trichinella* infection. Recently Kang et al. [143] showed that* T. spiralis*-induced Treg cells migrate to the inflammation site and suppress immune responses more effectively than non-parasite-induced Treg cells. The authors found that Foxp3+ T cells derived from* T. spiralis*-infected mice migrated to inflammation sites in the lung and expressed higher levels of Treg cell homing receptors (CCR5 and CCR9) and activation markers (Klrg1, Capg, GARP, Gzmb, and OX40) compared to Foxp3+ T cells from uninfected mice.

Infection with* T. spiralis* can apparently confer protection against EAAI by inducing a regulatory network where Treg cells may play an important role. However, other cell populations such as regulatory B cells and alternatively activated macrophages might also be involved [12, 144, 145]. Altogether, studies using animal models indicate that* T. spiralis* infection can protect against EAAI and that the mechanism of action involves Treg cell. The magnitude of this protective effect increases as* T. spiralis* infection progresses from the intestinal or acute phase to the muscle or chronic phase of infection. Although it is clear from studies using animal models that* T. spiralis* can induce an immunomodulatory network that may allow the host to deal with various hyperimmunity-associated disorders [12, 146], there are no human epidemiological studies addressing the inverse association between infection with this particular helminth and immunopathologies. Studies in humans are therefore required addressing this association in addition to identifying the parasitic antigens mediating immunosuppression.

### 5.3. Immunomodulation of Malignancy

Besides the here reported ability of* T. spiralis* to modulate allergic and autoimmune disorders there is a strong indication that this helminth exhibits also an antitumor effect. It was in the second part of the previous century that* T. spiralis* has been recognized for the first time as a nematode that can negatively influence a tumor growth and prolong the life span [147, 148]. However, the observed potential of* Trichinella* to affect tumor development was not investigated until recently, when Wang et al. [13] showed that* T. spiralis* infection as well as treatment of mice with mixture of crude extracts from adult parasite and newborn larvae can slow down or even inhibits the progression of tumors induced by different tumor cell lines. Also, the authors demonstrated strong antiproliferative and high proapoptotic influence of* T. spiralis* antigens on two different cell lines* in vitro* (K562 and H7402). Even in case of very aggressive tumor cell lines like B16 melanoma,* T. spiralis* infection succeeds to reduce not only the growth but also malignant cell dissemination [149]. In line with these findings our investigation confirmed the capacity of* T. spiralis* infection to restrain B16 melanoma development in C57Bl/6 mice [150]. Findings indicate that infection slows down tumor necrosis and slightly increases apoptosis* in vivo* and that* T. spiralis* ES L1 exhibited mild antisurvival and proapoptotic impact on B16 melanoma cells* in vitro*. It is obvious that powerful* Trichinella* antimalignance capacity does not rely only on necrosis and apoptosis provoked by its presence, indicating that other mechanisms through which infection or parasite products manipulate the tumor establishment and expansion are involved and are yet to be discovered. Gong et al. [151] investigated the presence of myeloma-associated antigens in* T. spiralis* and they have found that tropomyosin, a component of* T. spiralis* myofibrils, is the molecule that possesses antitumor effect, as well as the role in eliciting cross-reactive immunity. Treatment of mice with* T. spiralis* crude antigens, ES L1 antigens, tropomyosin, and the infection with 400 L1 larvae had very similar effect on tumor growth; that is, all these treatments inhibited the development of myeloma SP2/0. Recent research included recombinant* T. spiralis* protein A200711 that has proapoptotic effect on H7402 cell line and hence it was proposed as a therapeutic agent in hepatocellular carcinoma treatment [152]. These observations indicate that antitumor effects of parasite infection may be exhibited through the influence on the rate of malignancy and suppression of tumor growth and dissemination.

The here discussed experimental models provide insight into the complex host-parasite interactions and the immunoregulatory mechanisms induced by* T. spiralis* that could be involved in amelioration of autoimmune, allergic, and malignant diseases. Better understanding of these mechanisms, as well as identifying the parasite-molecules with immunomodulatory properties, could open future perspectives for new therapeutic approaches in the treatment of these diseases.
